# Obituary of Mrs. Gillett

**Published:** 1869-04

**Authors:** 


### OBITUARY.

Mrs. E. J. Gillett, wife of Prof. E. J. Gillett, M.D., D.D., died very suddenly, at her residence, near Fon du Lac, Wis., on Sunday, March 28th. Mrs. G. was in her usual health at 7 a.m., and died before 8 o'clock. This sudden dispensation of Providence has deprived a husband of a devoted wife, a family of an affectionate mother, and the church of a consistent christian. Her numerous friends in this city, where she was so well known, tender their heartfelt sympathies to the family and friends in this their hour of severe trial.

